# Computational machine learning analysis and validation for estimation of viscosity of ionic liquids versus temperature and composition

**DOI:** 10.3389/fchem.2026.1770244

**Published:** 2026-03-12

**Authors:** Yi Liu, Haoran Chen, Dong Li, Chonghao Bi, Javed Iqbal

**Affiliations:** 1 School of Computer and Artificial Intelligence, Beijing Technology and Business University, Beijing, China; 2 College of Engineering, China Agricultural University, Beijing, China; 3 Department of Applied Sciences, Faculty of Applied Sciences and Technology, Technological University of the Shannon: Midlands Midwest, Limerick, Ireland

**Keywords:** adaptive LASSO, ionic liquids, machine learning, spline regression, twin support vector regression

## Abstract

This study centers on predicting the viscosity of ionic liquid systems utilizing advanced regression models and a dataset comprising 8,500 entries. The input variables include categorical features (Cation and Anion) which represent the structure of ionic liquid and numerical variables (Temperature, T, and xIL). The data underwent several preprocessing steps, including Leave-One-Out encoding for categorical variables, Isolation Forest for outlier removal, and Min-Max method for normalization. Four regression models were implemented: Spline Regression (SPR), Twin Support Vector Regression (TSVR), Adaptive Lasso (ALASSO), and Neural Oblivious Decision Ensembles (NODE). Hyperparameters were optimized using the Firefly Algorithm. The NODE model indicated the best fitting amongst others, offering the highest cross-validation R^2^ of 0.99536 (±0.00124), training R^2^ of 0.99728, and test R^2^ of 0.99721, with the lowest test RMSE (0.0031499) and test MAE (0.0022219). The SPR model followed closely, with a cross-validation R^2^ of 0.96940 (±0.00303), test RMSE of 0.01393, and test MAE of 0.003869. TSVR showed moderate performance with a cross-validation R^2^ of 0.85577 and test RMSE of 0.01752, while ALASSO was the least effective, with a cross-validation R^2^ of 0.78169 and test RMSE of 0.02507. This study highlights the importance of robust preprocessing and identifies the NODE model as the most accurate and reliable tool for predicting viscosity in complex ionic liquid datasets.

## 1 Introduction

There is a momentum in growing utilization of Ionic Liquids (ILs) in different areas, and they are replacing organic solvents due to attractive properties such as recyclability, and the environmentally friendly nature of ILs. These novel materials can be used for various applications including separation, energy production, environmental science, green chemistry etc. (Akhlaq and Uroos, 2025; Baylan et al., 2024; Danarti et al., 2024; Usman et al., 2025; Xing et al., 2025; Zhang et al., 2025). As such, preparation and analysis of properties of ILs is of vital importance for development of these materials in manufacturing and separation applications. Understanding the behavior of ILs can help one to tailored-design the ILs with desired properties (Huwaimel et al., 2024).

Among the properties of ILs, viscosity is important as it can influence the application of ILs in processes (Prabhune et al., 2024). Description of ILs viscosity can be carried out via computational models to reduce the time and costs of measurements, while a vast majority of ionic liquids can be analyzed via computational models (Lei et al., 2023; Lei et al., 2024; Ma et al., 2025). Various studies have been reported on the evaluation of ILs properties via computational models. Huwaimel et al. (2024) investigated the viscosity of wide range of ILs via machine learning (ML) models. They utilized ML algorithms of Random Forest, Gradient Boosting, and XGBoost to correlate viscosity of ILs to structure, temperature and composition in IL-water mixtures. Indeed, the composition and temperature were taken into account as numerical inputs. Ran et al. (2024) conducted modeling properties of ILs via several machine learning methods such as K-Nearest Neighbors, Kernel Ridge Regression, and Lasso Regression. The fitting accuracy was reported to be excellent with the highest R^2^ value of 0.91(Test score) using K-Nearest Neighbors model. Recently, Han developed ML techniques for correlation of viscosity of ILs to temperature and concentration (Han, 2025). Three regressive models including Gaussian Process Regression, Multilayer Perceptron, and Support Vector Regression were employed for estimation of viscosity, and the best fit was obtained using Gaussian Process Regression model.

Despite development and utilization of multiple ML models for estimation of ILs viscosity, more ML algorithms should be explored and validated to find the best one with robust performance in accurate estimation of ILs viscosity (Basarkar and Dey, 2022; Chen et al., 2024). Thus, this research gap is addressed in this study via development of new ML models for description of ILs viscosity based on input parameters. ML models have been used due to their robustness and power in calculation of ILs properties such as viscosity. Emerging as a useful tool for solving challenging modeling tasks in many disciplines is machine learning. It reveals data-based trends and relationships. Its capacity to control both linear and non-linear relationships makes it especially helpful for multi-variable systems in outcome prediction. In this work, ionic liquid system viscosity is predicted using ML methods. Combining numerical and categorical inputs provides the foundation of this prediction (Jordan and Mitchell, 2015). The structure of ILs (e.g., anion and cation type) is the categorical input, while composition and temperature are the numeric inputs.

The methods selected for this work include Spline Regression (SPR), Twin Support Vector Regression (TSVR), Neural Oblivious Decision Ensembles (NODE), and Adaptive Lasso (ALASSO). These models were chosen for their distinct advantages. Using piecewise polynomial functions, SPR is great at modeling non-linear trends which means it can handle the complex relationships in the dataset. TSVR provides flexibility and scalability through its dual hyperplane approach and kernel functions, enabling it to handle non-linear data effectively. ALASSO, with its adaptive regularization weights, enhances feature selection and improves interpretability by prioritizing the most relevant features. NODE utilizes differentiable oblivious decision trees within a neural network framework, offering superior predictive accuracy and generalization for tabular data through end-to-end gradient-based optimization.

This work develops the field by offering a methodical approach combining strong data preprocessing techniques with modernized ML models. Data quality and consistency are maintained in part by preprocessing actions including Min-Max scaling for normalizing, Isolation Forest for outlier removal, and Leave-One-Out encoding of categorical variables. Furthermore, shown in the paper is the hyperparameter optimization application of the Firefly Algorithm.

This study introduces a distinctive approach by integrating advanced machine learning techniques, specifically Neural Oblivious Decision Ensembles (NODE), with a comprehensive dataset of ionic liquid systems, an area underexplored in prior research. Unlike earlier studies that predominantly relied on traditional regression models, our work harnesses NODE to achieve enhanced predictive accuracy and generalization. Furthermore, we employ the Firefly Algorithm for hyperparameter optimization, a novel strategy that bolsters the robustness and reliability of our models. This unique combination of cutting-edge methods distinguishes our research and advances computational modeling of ionic liquid’s properties.

## 2 Dataset and visualization

The dataset contains 8,500 rows and includes numerical inputs, i.e., T(K) and xIL(mol%) in addition to categorical inputs like Cation and Anion. Viscosity (Pa·s) serves as the output in this analysis (Han, 2025). The dataset is taken from previous publication, and more details regarding the notations and measurements can be found elsewhere (Chen et al., 2022). The ionic liquid with different functional groups such as imidazolium, ammonium, and pyridinium are studied in this work. The data covers a wide range of ILs with 8 various cations (Chen et al., 2022; Huwaimel et al., 2024). The mixture of IL and water was considered under different conditions (Han, 2025).

**FIGURE 1:**
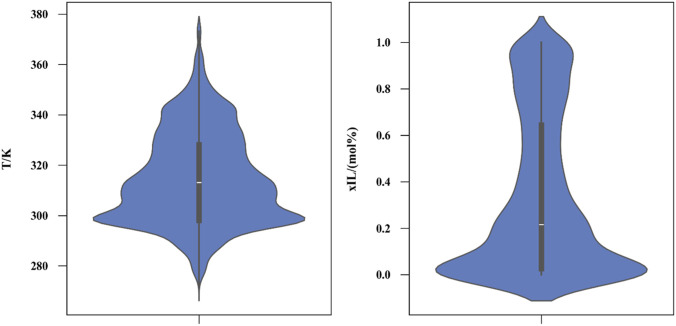
Violin plot of numeric inputs.

Figure 1 presents a violin plot of the numerical inputs derived from the raw dataset. Typically, a violin plot combines a box with a kernel density plot to illustrate the probability density of the data. In this context, the numerical inputs likely correspond to variables such as temperature (T in Kelvin) and concentration (xIL in mol%).

To prepare the dataset for analysis, several preprocessing steps were applied. The goal of these steps was to deal with both categorical and numerical data, eliminate any outliers, and make the features more similar, resulting in improved modeling performance.

Initially, the categorical features (Cation and Anion) were converted into numerical values using the Leave-One-Out (LOO) encoding method. This method replaces each category with the average of the target variable while excluding the current row. This technique effectively maintains the relationship between the categorical variables and the target variable without introducing data leakage (Seger, 2018).

Next, outliers in the dataset are spotted using the Isolation Forest algorithm, and then they are all deleted from further analysis. This approach detects data points that are significantly different from other data points. By removing these anomalies, the dataset became more consistent, which improves the reliability of the analysis and modeling (Cheng et al., 2019; Liu et al., 2008).

Finally, the numerical features were normalized using Min-Max scaling. This technique adjusts the range of each numerical feature to lie between 0 and 1. Normalization ensures that all features have the same scale, making them easier to compare and more suitable for ML algorithms (Ambarwari et al., 2020).

## 3 Methodology

The methodology employed in this study combines robust data preprocessing, model implementation, and optimization techniques to accurately predict viscosity based on categorical and numerical inputs. The dataset was first preprocessed to handle categorical features through Leave-One-Out encoding, remove outliers using the Isolation Forest algorithm, and normalize numerical features via Min-Max scaling. This preprocessing ensured a clean, standardized dataset suitable for modeling.

Regression models—SPR, TSVR, NODE, and ALASSO—were implemented. These models were chosen for their ability to capture non-linear relationships, enhance feature selection, and handle complex data distributions. SPR utilized flexible cubic splines to model non-linear trends, TSVR employed dual hyperplanes with kernel functions for scalability and efficiency, and ALASSO improved feature selection with adaptive regularization weights.

### 3.1 Spline regression (SPR) model

SPR is a powerful statistical technique used to model complex, challenging correlations between independent (input) and dependent (output) variables. By dividing the range of the independent variable into segments and fitting separate polynomial functions to each segment, spline regression captures the intricacies of data that linear models often miss. This flexibility is achieved through the use of knots, which are predefined points where the polynomial pieces connect. The resulting piecewise function is smooth and continuous, allowing for a more accurate representation of the underlying data structure (Marsh and Cormier, 2001).

A common form of spline regression involves cubic splines, which are piecewise cubic polynomials defined over intervals determined by the knots. The individual cubic spline equation with just one knot at a location *g* can be expressed as follows (Ajao et al., 2023):

$$y_{i}=\left\{\begin{array}{l} \beta_{01}+\beta_{11}z_{i}+\beta_{21}z_{i}^{2}+\beta_{31}z_{i}^{3}+\epsilon_{i}\text{ if }z_{i}<g\\ {\;\beta }_{02}+\beta_{12}z_{i}+\beta_{22}z_{i}^{2}+\beta_{32}z_{i}^{3}+\epsilon_{i}\text{ if }z_{i}\geq g\; \end{array}\right.$$

In this equation, $y_{i}$ represents the dependent variable that we aim to predict or explain. The variable $z_{i}$ is the independent variable, which serves as the input for the model. The knot *g* is a specific value of $z_{i}$ that determines where the piecewise function changes its form. The coefficients $\beta_{0j},\beta_{1j},\beta_{2j}$ , and $\beta_{3j}$ are parameters to be estimated from the data, where *j = 1, 2* corresponds to the segments of the spline before and after the knot *g*, respectively. Finally, $\epsilon_{i}$ denotes the error term, capturing the variability in $y_{i}$ that is not explained by the model. This formulation allows spline regression to effectively model non-linear relationships by using different polynomial expressions in each segment defined by the knot.

### 3.2 Twin support vector regression (TSVR)

TSVR is an advanced ML technique tailored for regression tasks. Unlike traditional Support Vector Regression (SVR), which formulates a single optimization problem, TSVR divides the problem into two smaller, independent sub-problems (Khemchandani and Chandra, 2017). These sub-problems aim to generate two non-parallel hyperplanes, each closer to one set of data points while maintaining an appropriate margin from the other set. This division enhances the efficiency and flexibility of the model, particularly for handling complex datasets with non-linear relationships (Tanveer et al., 2022; Tomar and Agarwal, 2015).

The TSVR approach introduces two regression hyperplanes, represented mathematically as follows:

$$w_{1}^{T}x+b_{1}=0\text{ and }w_{2}^{T}x+b_{2}=0$$

Here, $w_{1}$ and $w_{2}$ stand for the weight vectors, $b_{1}$ and $b_{2}$ are the bias terms, and *x* denotes the input features. The optimization objectives for these hyperplanes are to minimize their respective distances from one subset of the data points while maximizing the distance from the other. This dual optimization makes TSVR computationally efficient compared to standard SVR, especially for large datasets (Tanveer et al., 2022).

### 3.3 Adaptive Lasso (ALASSO) regression model

Adaptive Lasso Regression is designed to enhance variable selection and improve estimation accuracy. It represents an advancement over the standard Lasso (Least Absolute Shrinkage and Selection Operator) method. This approach addresses certain limitations of standard Lasso by applying adaptive weights to the penalty terms. These limitations include insufficient shrinking of significant coefficients and challenges in accurately identifying the true subset of relevant variables (Ivanoff et al., 2016; Tomar and Agarwal, 2015).

The Adaptive Lasso determines the regression coefficients by solving an optimization problem. This problem seeks to achieve the optimal balance between the model’s fit and the extent to which the coefficients are penalized. Mathematically, the coefficients are determined as (Zou, 2006):

$$\hat{\beta }=\arg\min_{\beta }\left\{\frac{1}{2n}\sum_{i=1}^{n}{\left(y_{i}-x_{i}^{T}\beta \right)}^{2}+\lambda \sum_{j=1}^{p}w_{j}\left|\beta_{j}\right|\right\}$$

In this equation, $y_{i}$ represents the observed response variable for the *i*th point, while $x_{i}$ denotes the corresponding vector of predictor variables (input features). The term $\beta_{j}$ stands for the regression coefficient for the *j*th predictor variable, while λ refers to the regularization coefficient. The weights $w_{j}$ are adaptive and assigned to each coefficient $\beta_{j}$ . This assignment allows for the adjustment of the penalty based on the variable’s importance. Usually, an Ordinary Least Squares (OLS) or Ridge regression model is used to get an approximate estimation of the coefficients, which is then employed for determining the weights. Specifically, the weights are defined as $w_{j}=\frac{1}{{\left|\hat{\beta_{j}}\right|}^{\gamma }}$ , where $\hat{\beta_{j}}$ represents the initial estimate of the j-th coefficient, and γ> 0 serves as a tuning parameter that regulates the extent to which the weights adapt to the coefficients.

Because the weights are adaptive, variables with larger initial coefficients receive penalties less. This lets the model keep important predictors and get free of less important ones. This weighting mechanism gives Adaptive Lasso the oracle property, implying it can detect the true subset of variables and provide nearly unbiased estimates of their coefficients.

### 3.4 Neural oblivious decision ensembles (NODE)

NODE represents an innovative machine learning approach, useful for tabular datasets such as those encountered in ionic liquid viscosity prediction. Introduced in (Popov et al., 2019), NODE integrates the structured decision-making capabilities of decision trees with the flexibility of neural networks, creating a differentiable ensemble model optimized via gradient-based methods (Ghazwani and Hani, 2025).

In NODE, the core component is a collection of oblivious decision trees (ODTs), where each tree employs identical feature splits and thresholds at every level across all branches (Ghazwani and Hani, 2025). This uniformity simplifies the tree structure, reducing overfitting while maintaining predictive power. Each ODT assigns input data to a leaf node, which contains a learnable weight corresponding to the predicted viscosity value. The ensemble aggregates the outputs of all trees, typically through a weighted sum, to produce the final regression output. Mathematically, for an input vector x , the NODE prediction can be expressed as Ghazwani and Hani (2025), Popov et al. (2019):

$$y=\sum_{t=1}^{T}w_{t,l\left(x\right)}\cdot I_{l\left(x\right)}$$

where T is the number of trees, $w_{t,l\left(x\right)}$ is the weight of the leaf node $l\left(x\right)$ for tree t to which input x is assigned, and $I_{l\left(x\right)}$ is an indicator function that activates the corresponding leaf.

The differentiability of NODE allows it to be trained end-to-end using gradient descent, leveraging deep learning frameworks. This enables the model to optimize both the tree structure (e.g., feature thresholds) and leaf weights simultaneously, enhancing its ability to capture complex patterns in the data. For the ionic liquid dataset, NODE effectively handles the combination of categorical features (Cation and Anion) and numerical inputs (Temperature and xIL), making it well-suited for modeling non-linear relationships in viscosity prediction of ILs.

### 3.5 Firefly optimization algorithm (FFA)

**FIGURE 2:**
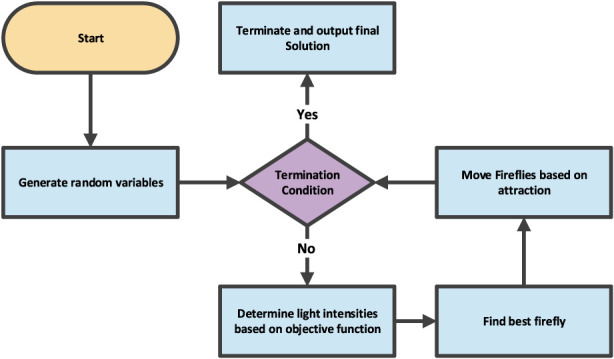
The procedure for FFA model (Moin et al., 2025).

FFA draws inspiration from the natural behavior of fireflies for solving problems with optimization tasks. In this study, we employed this technique for hyper-parameter tuning. Within the algorithm, each firefly symbolizes a randomly generated solution. The brightness or luminosity of a firefly indicates the quality of the solution, as calculated by the objective function (Yang et al., 2012). The method runs iteratively until it converges. Adjusting variables like the degree of the domain and the number of fireflies helps one change the rate of convergence. Figure 2 offers an overview of the FFA algorithm’s operational steps (Moin et al., 2025; Yang, 2013).

The FFA was employed to optimize the mean R^2^ from 5-fold cross-validation for hyperparameter tuning. This method yields better results than conventional grid search because it can explore a more diverse set of hyperparameter values, rather than being restricted to predefined fixed values. By continuously searching the hyperparameter space, FFA increases the chances of finding superior hyperparameter configurations, thereby enhancing the model’s performance (Moin et al., 2025).

## 4 Results and discussion

A split of 85%–15% was used to separate the dataset into training and testing subsets for this study and developing the ML models. This allocation ensured that a significant portion of the data was available for model training while leaving a separate subset for a thorough evaluation of the model’s accuracy. The results presented here include metrics from the training and testing phases, as well as cross-validation scores to assess the reliability and generalizability of the models. Detailed comparisons of the optimized models are also provided, highlighting their strengths in predictive accuracy and error minimization. The models developed here are compared and discussed to find out which model performs the best in estimation of ILs viscosity.

In both accuracy and generalization, the NODE model proved better than the others according to the results and the statistical analyses. Table 1 highlights the NODE model as the best performer, achieving the highest cross-validation R^2^ of 0.99536 ± 0.00124, training R^2^ of 0.99724, and test R^2^ of 0.99718. These results demonstrate NODE’s exceptional predictive accuracy and robust generalization on unseen data. In comparison, the SPR model also performed strongly, with a cross-validation R^2^ of 0.96940 ± 0.00303, training R^2^ of 0.98933, and test R^2^ of 0.95037, but it was outperformed by NODE. The TSVR model showed moderate performance, with a cross-validation R^2^ of 0.85577 ± 0.02385, R^2^ of 0.94680 (training), and test R^2^ of 0.92107. The ALASSO model exhibited the weakest performance, with a cross-validation R^2^ of 0.78169 ± 0.00469, training R^2^ of 0.85977, and test R^2^ of 0.83837.

**TABLE 1:** R^2^ values of the optimized ML models in this study.

Model	K-fold (k = 5) CV R^2^	Final train R^2^	Final test R^2^
ALASSO	0.78169 ± 0.00469	0.85977	0.83837
SPR	0.96940 ± 0.00303	0.98933	0.95037
TSVR	0.85577 ± 0.02385	0.94680	0.92107
NODE	0.99536 ± 0.00122	0.99728	0.99721

Table 2 corroborates these findings through error metrics. NODE obtained the lowest fitting errors, with a training RMSE of 2.9137E-03, test RMSE of 3.1499E-03, training MAE of 1.49352E-03, and test MAE of 2.22190E-03, underscoring its superior precision. SPR followed, with a training RMSE of 5.7249E-03, test RMSE of 1.3893E-02, training MAE of 1.91650E-03, and test MAE of 3.86932E-03. TSVR’s performance was intermediate, with a training RMSE of 1.2785E-02, test RMSE of 1.7521E-02, training MAE of 4.16304E-03, and test MAE of 5.41338E-03. ALASSO recorded the highest errors, with a training RMSE of 2.0756E-02, test RMSE of 2.5072E-02, training MAE of 1.06792E-02, and test MAE of 1.21048E-02.

**TABLE 2:** Error metrics of the optimized ML models.

Model	Training		Test	
	RMSE	MAE	RMSE	MAE
ALASSO	2.0756E-02	1.06792E-02	2.5072E-02	1.21048E-02
SPR	5.7249E-03	1.91650E-03	1.3893E-02	3.86932E-03
TSVR	1.2785E-02	4.16304E-03	1.7521E-02	5.41338E-03
NODE	2.9137E-03	1.49352E-03	3.1499E-03	2.22190E-03

**FIGURE 3:**
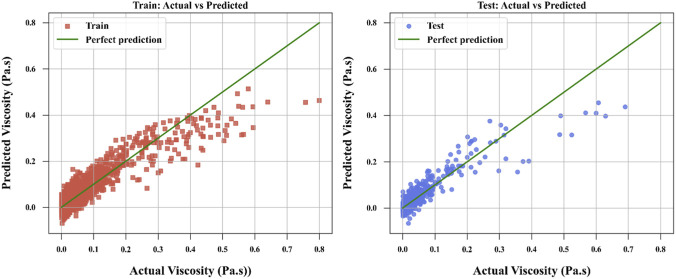
ALASSO model to compare actual and calculated values.

**FIGURE 4:**
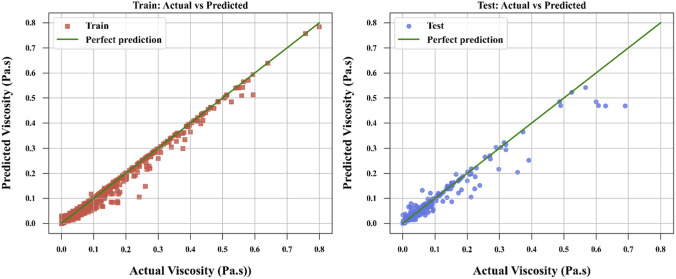
SPR model to compare actual and calculated values.

**FIGURE 5:**
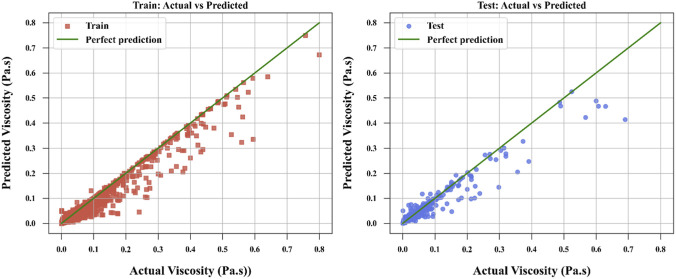
TSVR model to compare actual and calculated values.

**FIGURE 6:**
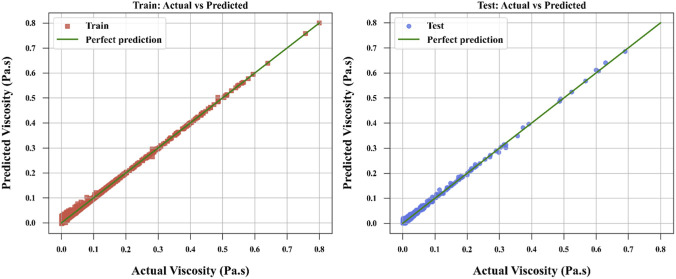
NODE model to compare actual and calculated values.

Figures 3–6 provide further visual evidence supporting these findings. Parity plots comparing actual and predicted values illustrate that the NODE model (Figure 6) aligns most closely with the ideal parity line, demonstrating its exceptional accuracy and confirming its position as the best-performing model. The accuracy for NODE model developed and optimized in this study is shown to be better than the models developed by Huwaimel et al. (2024) for correlation of ILs’ viscosity which reported the best RMSE of 4.7240E-03 for the same dataset using Random Forest (RF) model. The comparative analysis is provided in Table 3 between this work and the best model developed by Huwaimel et al. (2024).

**TABLE 3:** Comparative analysis of the results obtained in this study with previous study reported in Huwaimel et al. (2024).

Model	Training		Test	
	RMSE	R^2^	RMSE	R^2^
RF [reported by Huwaimel et al. (2024)]	4.7240E-03	0.99736	9.0301E-03	0.99718
NODE (this study)	2.9137E-03	0.99728	3.1499E-03	0.99721

The SPR model (Figure 4) also shows strong alignment with the parity line, reflecting its high predictive accuracy, though slightly less precise than NODE. In contrast, the ALASSO model (Figure 3) exhibits more significant deviations from the parity line, indicating poorer predictive performance. TSVR (Figure 5) performs better than ALASSO but does not match the precision of SPR or NODE, as its deviations from the parity line are more pronounced than those of SPR and substantially greater than those of NODE.

Overall, the analysis of metrics and figures clearly establishes NODE as the best one in terms of both predictive accuracy and generalization ability, making it the most suitable choice for this dataset. The SPR model, while highly effective, is outperformed by NODE, as evidenced by the closer alignment in Figure 6.

**FIGURE 7:**
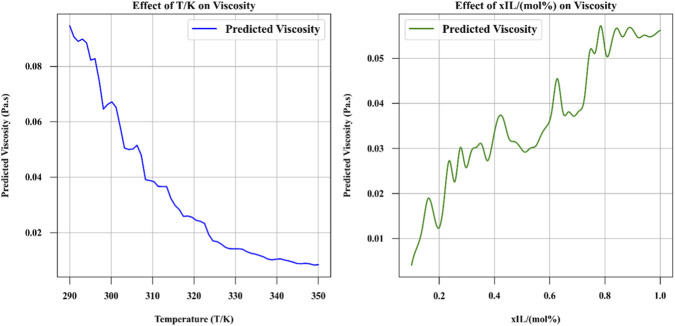
The effect of numeric inputs on viscosity (fixed T = 303.15, xIL = 0.766, Cation = [C4mim] and Anion = [C(CN)3]).

**FIGURE 8:**
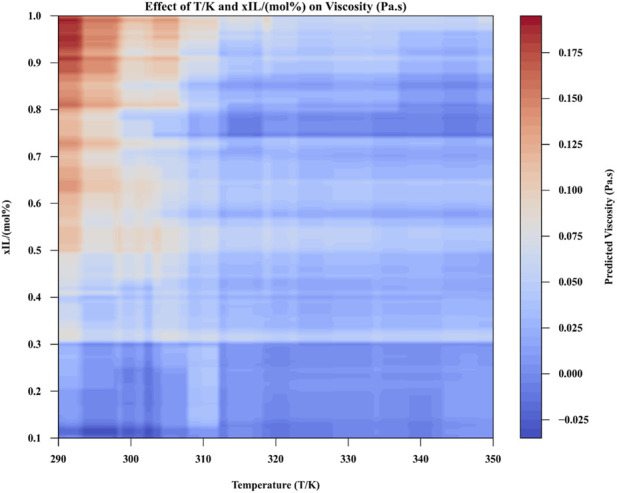
Contour plot of predicted viscosity based on two numeric inputs (fixed Cation = [C4mim] and Anion = [C(CN)3]).

Figures 7, 8 provide insights into the effect of numerical inputs on viscosity and how the model predicts viscosity under different conditions (Han, 2025). Figure 7 illustrates the effect of varying numeric inputs on viscosity while keeping specific conditions fixed (T = 303.15 K, xIL = 0.766, Cation = [C4mim], and Anion = [C(CN)3]). It highlights how changes in the selected variables influence the output viscosity (Huwaimel et al., 2024).

Figure 8 is a contour plot showing the predicted viscosity as a function of two numerical inputs, with other categorical features (Cation and Anion) held constant. This visualization provides a two-dimensional map of viscosity values across a range of input variables, making it easier to identify patterns and gradients (Huwaimel et al., 2024).

**FIGURE 9:**
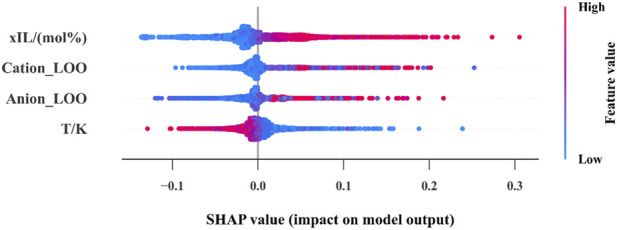
SHAP plot for the variables.

SHAP (SHapley Additive exPlanations) explains model predictions by showing how each feature contributes to the output (Bowen et al., 2020). Figure 9 shows a summary plot that ranks features by how important they are in the predictive model. The x-axis shows the impact magnitude (SHAP value), and the colors show the feature values (high or low).

The plot reveals that temperature (T) and xIL are the most significant factors influencing viscosity. While temperature shows a mixed effect, high xIL values raise viscosity. Small but significant effects were observed from the cation and anion categorical variables. This indicates that the model effectively captures essential features, contributing to precise viscosity predictions.

## 5 Conclusion

In In this study, multiple regression models, including ALASSO, TSVR, NODE, and SPR, were employed to predict the viscosity of ionic liquid systems using a combination of categorical (Cation and Anion) and numerical (Temperature, T, and xIL) data. To ensure model robustness, the dataset of 8,500 entries was meticulously preprocessed through Leave-One-Out encoding for categorical variables, Isolation Forest for outlier removal, and Min-Max normalization for numerical features. The NODE model outperformed all others, achieving the highest cross-validation R^2^ of 0.99536 (±0.00124), training R^2^ of 0.99728, and test R^2^ of 0.99721, with the lowest test RMSE of 0.0031499 and test MAE of 0.0022219. These results highlight NODE’s exceptional ability to capture complex non-linear relationships and generalize effectively on unseen data, attributed to its differentiable ensemble of oblivious decision trees optimized via gradient-based methods. The SPR model followed closely, with a cross-validation R^2^ of 0.96940 (±0.00303), test R^2^ of 0.95037, test RMSE of 0.01393, and test MAE of 0.003869, demonstrating strong performance in modeling non-linear trends through piecewise polynomial functions.

TSVR exhibited moderate performance with a cross-validation R^2^ of 0.85577, test R^2^ of 0.92107, test RMSE of 0.01752, and test MAE of 0.005413, leveraging its dual hyperplane approach but falling short of NODE and SPR in accuracy and dependability. ALASSO was the least effective, with a cross-validation R^2^ of 0.78169, test R^2^ of 0.83837, test RMSE of 0.02507, and test MAE of 0.012104, despite its adaptive regularization for feature selection.

Additionally, SHAP analysis revealed that temperature (T) and xIL were the most influential features, with NODE effectively capturing their complex interactions. These results underscore the power of NODE as the most reliable and accurate tool for viscosity prediction in ionic liquid systems, advancing computational modeling through its integration of neural network optimization with structured decision-making, while SPR remains a robust alternative for handling non-linear data trends.

## Data Availability

The original contributions presented in the study are included in the article/supplementary material, further inquiries can be directed to the corresponding author.
